# A cross-sectional study of physical activity participation among adults with chronic conditions participating in a digital health program

**DOI:** 10.1177/2055207619880986

**Published:** 2019-10-20

**Authors:** Renae L Smith-Ray, Nima Nikzad, Tanya Singh, Jenny Z Jiang, Michael S Taitel, Giorgio Quer, Jean Cherry, Steven R Steinhubl

**Affiliations:** 1 Walgreens Center for Health and Wellbeing Research, Walgreen Co., Deerfield, Illinois, USA; 2 Department of Digital Medicine, Scripps Translational Science Institute, La Jolla, California, USA; 3 Experian DataLabs, San Diego, California, USA

**Keywords:** Wearable devices, mobile technologies, physical activity, chronic disease

## Abstract

**Objective:**

Many American adults are insufficiently active. Digital health programs are designed to motivate this population to engage in regular physical activity and often rely on wearable devices and apps to objectively measure physical activity for a large number of participants. The purpose of this epidemiological study was to analyze the rates of physical activity among participants in a digital health program.

**Method:**

We conducted a cross-sectional study of participants enrolled in a digital health program between January 2014 and December 2016. All activity data were objectively collected through wearable devices.

**Results:**

Participants (*n *=* *241,013) were on average 39.7 years old and 65.7% were female. Participants walked on average 3.72 miles per day. Overall, 5.3% and 21.8% of participants were being treated with diabetes and cardiovascular medications respectively, but these rates varied across young, middle and older adults. Participants of all ages being treated with cardiovascular and/or diabetes medications walked significantly less than those not being treated for these conditions.

**Conclusion:**

The feasibility of using a large database containing data from consumer-grade activity trackers was demonstrated through this epidemiological study of physical activity rates across age and condition status of participants. The approach and findings described may inform future research as the information age brings about new opportunities to manage and study massive amounts of data generated by connected devices.

## Introduction

The benefits of regular physical activity are both extensive and well established. The Centers for Disease Control and Prevention (CDC) recommends that each week adults engage in at least 150 minutes of moderate-intensity aerobic activity or 75 minutes of vigorous-intensity aerobic activity.^1^ Examples of moderate-intensity physical activities include brisk walking, bicycling <10 mph on level ground, recreational swimming, leisurely canoeing/kayaking, and gardening or yardwork. Examples of vigorous-intensity activities include running, bicycling >=10 mph, aerobic dancing, swimming laps, jumping rope, and most competitive sports.^1^ Adults who engage in the recommended weekly amount of physical activity reduce their risk of developing cardiovascular disease (CVD) by 17% and have a 23% lower risk of CVD-attributable mortality.^1^ Compared to those with low physical activity, those who engaged in regular physical activity had a 35% decreased risk of developing type 2 diabetes.^2^ Regular physical activity also decreases obesity, reduces risk of various forms of cancer, improves depressive symptoms and improves cognitive health.^3–5^ While cardiovascular fitness is beneficial to all, it is particularly important for those with CVD and type 2 diabetes as it is associated with improved insulin sensitivity, improved blood pressure, and decreased inflammation.^6^ The positive impact of physical activity on health is so convincing that exercise has been called both a “magic bullet” for preventing obesity and CVD and one of the most effective approaches to promote successful aging.^7–9^

Physical inactivity places a substantial burden on population health.^10^ In 2014, 27.5% of US adults age 50+ were physically inactive.^11^ The prevalence of physical inactivity was substantially higher among adults with coronary heart disease (37.2%) and diabetes (38.4%).^11^ However, adults with CVD self-reported even lower rates of physical activity; between 39.7% and 53.8% of adults with CVD report engaging in physical activity.^12^ Those with diabetes also recorded lower accelerometer-measured total activity count compared to participants with normal glucose levels or prediabetes.^13^ Physical activity participation rates decline even further in the presence of more than one chronic condition.^14^ A systematic review found that even a modest increase in physical activity among previously inactive adults with CVD or diabetes results in substantial improvements in morbidity and mortality.^1^

Until recently the most common methods used to collect physical activity data involved either self-reported data, which can be inaccurate,^15,16^ or research-grade objective activity measures, such as accelerometers, which can be costly.^17^ The prevalence of consumer-grade digital activity trackers has increased dramatically in recent years.^18^ Wearable fitness trackers, one of the fastest growing consumer technologies, are estimated to be owned by 20% of American households.^18^ By 2021, the number of those using wearable fitness trackers is expected to be over 75 million, about double the current users.^19^

Although wearable accelerometers remain the gold standard for objective physical activity measurement in research, a clinical study that examines accelerometer-derived physical activity rates of hundreds of thousands of users would be unmanageable. The broad adoption of consumer-grade activity trackers and increased access to large amounts of data provide a novel opportunity to examine population health rates. The validity of many consumer-grade activity monitors has been demonstrated by capturing activity readings similar to those captured by research-grade devices.^20,21^ With careful data cleaning and analysis, massive amounts of data from connected devices, such as consumer-grade activity trackers, can be used in a research capacity to explore rates in population health.

The primary purpose of this study is to demonstrate the viability of using a large database containing data from consumer-grade activity trackers to conduct a study of population-level physical activity rates. The secondary purpose of this study is to conduct an epidemiological analysis of physical activity rates across age and condition status of participants in a digital health program, Balance Rewards for healthy choices (BRhc). The study may inform future research as the information age brings about new opportunities to manage and study massive amounts of data generated by connected devices. The approach and findings described may become particularly useful as research examinations of these data become more common.

## Method

### Study design

We conducted a cross-sectional study of adult participants in the BRhc program. Physical activity and pharmacy data collected between January 2014 and December 2016 were analyzed retrospectively. Data were acquired from secure Teradata servers where pharmacy and BRhc program data is stored.

### Participants

Study participants are BRhc participants who are at least 20 years old and who reported at least 20 walk and/or run activities during the data collection period. When participants enroll into BRhc, they agree to allow their de-identified BRhc data to be used for the purpose of health research. This study was approved with a waiver of written informed consent by Quorum Review institutional review board (#30491). All BRhc participants between the years 2014 and 2016 who did not opt out of using their data for BRhc health research were included into this study. Data were captured by consumer-grade physical activity trackers and physical activity (running) apps connected to the BRhc participants’ account.

### Program description

A large, nationwide pharmacy chain, Walgreens, developed BRhc—an internet- and mobile application–based reward program to incentivize customers to be physically active. The BRhc program was launched in 2012 as an approach to incentivize patients to track health behaviors including physical activity, body weight, tobacco use, and blood glucose and blood pressure readings. The BRhc program allows participants to track their physical activity participation manually or by connecting a variety of consumer-grade activity trackers to their BRhc digital account.^22^ BRhc is available online to anyone whether or not they have prescriptions filled at Walgreens. BRhc participants accumulate balance rewards points by setting a goal (250 points) and by walking, running, cycling or other activities receiving 20 points per mile, up to 1000 points per month. Exercise distance, duration and intensity can be entered either manually or through a digitally connected (i.e. Bluetooth) tracking device that is linked to the participants’ BRhc account. Activity tracker brands available for linking include the Fitbit Flex, Charge HR, Surge, Blaze, Alta, One, Ultra, Zip and Force, Withings Pulse, Jawbone Up and Up24, BodyMedia Fit Link, Misfit Shine and Striiv devices. Apps linking to the BRhc program with exercise tracking include Lose It!, Runkeeper, WebMD, MyFitnessPal, Charity Miles and MapMyFitness. Multiple apps or devices can be linked and a reward of 250 points is given for linking each device or app.

### Study data

This study includes activity data recorded by BRhc users between January 2014 and December 2016. Data were captured by consumer-grade physical activity trackers and physical activity (running) apps connected to the BRhc participants’ account. Activity data includes total miles walked/run per day. A total of approximately 84 million objective measures of physical activity collected from wearable devices were considered in this study. BRhc also allows participants to enter activity data manually; however, these data were not included as it is susceptible to participant subjectivity. Data from smartphone-based activity trackers such as iPhone Health Kit and Google Fit which were also excluded, since it is possible to manually enter an activity also in these apps. Among the 84 million objective entries considered, data were excluded if the device had less than 1000 unique users and less than 10,000 unique measurements recorded (11%). Entries for participants who did not report their date of birth were also excluded (2%). Entries reported multiple times over the course of the day were excluded (3%). Activity records of ≥30 miles per day were excluded from the analysis (0.03%).

Some apps allow the user to specify the type of activity (i.e. walk, run, bicycle, etc.). In addition to reporting total steps per day, this study also reports on running activity captured from devices capable of specifying this activity type. These apps differ from wearable devices in that the user must open the app and begin tracking a new activity each time he/she engages in a run. These apps collect data only when a new exercise session is indicated; they do not track steps over the course of the day. Other activities were excluded from analysis due to the low frequency of data being reported.

Participants who recorded a mean ≥7500 steps per day (i.e. 3.75 miles) were categorized as sufficiently active. This cut-point is based on a review of 42 studies that assessed speed, metabolic equivalents and cadence.^23^ The authors translated the physical activity measurements into step counts associated with moderate and vigorous physical activity (MVPA). Based on these computations, it was concluded that a range of 7000–8000 is an adequate representation of sufficient daily MVPA.

### Chronic condition categorization

Condition categories were identified using pharmacy claims data during the study period. BRhc program users using online pharmacy services were identified and their pharmacy records were matched to their physical activity data. Pharmacy patients were assigned to the chronic condition category if they filled one or more prescriptions for a medication that is used to treat the condition. A licensed pharmacist identified medications based on their generic product identifiers (GPI) for each of the select conditions (see Table 1). Cardiovascular and diabetes condition categories were identified using GPI drug groups. Users were split into five groups: patients treated with cardiovascular medications, diabetes medications, cardiovascular and diabetes medications, other pharmacy patients, and non-pharmacy customers.

**Table 1.** Categorization of prescribed medications by condition-type.

Condition category	Drug groups included
Cardiovascular	Antihyperlipidemics, beta blockers, antihypertensives, hematopoietic agents, calcium channel blockers, diuretics, anticoagulants, cardiovascular agents (miscellaneous), antiarrhythmics, vasopressors, antianginal agents
Diabetes	Antidiabetics

### Statistical analysis

One-way analysis of variance (ANOVA) was used to assess differences in daily miles walked/run and daily miles run between age groups and cardiovascular and diabetes conditions. Risk ratios were computed to show risk of inactivity by chronic condition status within each age group. Binomial regression was used to analyze the association between cardiovascular and/or diabetes treatment and the dichotomous dependent variable of sufficient physical activity (mean distance per day ≥3.75 miles). Three dichotomous independent variables were created for the chronic condition statuses: cardiovascular only, diabetes only, and both cardiovascular and diabetes. Each group was compared to pharmacy patients who did not have an indication for cardiovascular or diabetes conditions during the study period. Effect of age on the association between condition status and physical activity was assessed through analysis of covariance. Risk ratios and 95% confidence intervals (CIs) were then computed from the resulting models. All analyses were conducted using SAS 9.4 and SciPy 0.19.1.

## Results

We analyzed data from a total of 241,013 BRhc participants who had at least 20 walking and/or running events. Participants were on average 39.7 years old, and 65.7% were female. Their physical activity was recorded on average for 290 days (median 209) during the period in which they were enrolled in the program, which was on average 419.2 days (median 345), corresponding to a fraction of 72.7% (82.8%) of active days. Physical activity data was collected from 14 different wearable devices and six different apps. Daily step count was captured for all study participants; however, only 7.2% of participants reported at least 20 runs.

The majority of participants (53.6%) had at least one prescription filled at Walgreens during the study period. Of these users, 42.7% had a prescription filled related to the management of a cardiovascular condition or diabetes during the study period (see Table 2).

**Table 2.** Average daily walking and running distance among Balance Rewards for healthy choices participants across age groups and chronic conditions.

	Walking and running				Running only			
Age group	*n*	% of total	Mean miles (SD)	95% CI	*n*	% of total	Mean miles (SD)	95% CI
Full cohort								
All users	241,013	100	3.72 (1.60)	3.71–3.72	17,133	100	3.71 (1.73)	3.68–3.73
Female sex	158,356	65.7	3.60 (1.52)	3.59–3.60	9530	54.6	3.58 (1.67)	3.54–3.62
Male sex	39,844	16.5	4.02 (1.74)	4.00–4.03	3511	20.5	4.03 (1.92)	3.97–4.09
Unstated sex	42,813	17.8	3.88 (1.67)	3.86–3.90	4272	24.9	3.75 (1.69)	3.70–3.80
Cardiovascular	42,558	17.7	3.43 (1.52)	3.42–3.45	2099	12.1	3.62 (1.71)	3.54–3.69
Diabetes	2903	1.2	3.26 (1.38)	3.21–3.31	78	0.5	3.10 (1.41)	2.79–3.41
Cardiovascular and diabetes	9802	4.1	3.01 (1.44)	2.99–3.04	243	1.4	3.35 (1.54)	3.15–3.54
Other prescriptions	74,012	30.7	3.72 (1.51)	3.71–3.73	5570	32.2	3.71 (1.68)	3.67–3.75
No prescription data	111,738	46.4	3.89 (1.66)	3.88–3.90	9323	53.8	3.75 (1.78)	3.71–3.79
20–29 y								
All users	52,068	21.6	3.66 (1.43)	3.65–3.67	3998	100	3.49 (1.66)	3.44–3.54
Female sex	34,045	65.4	3.55 (1.36)	3.54–3.57	2326	58.2	3.42 (1.56)	3.35–3.48
Male sex	6768	13.0	3.99 (1.58)	3.96–4.03	553	13.8	3.72 (1.82)	3.57–3.88
Unstated sex	11,255	21.6	3.78 (1.49)	3.76–3.81	1119	28.0	3.52 (1.76)	3.42–3.62
Cardiovascular	5277	10.1	3.37 (1.33)	3.34–3.41	279	7.0	3.45 (1.56)	3.27–3.63
Diabetes	709	1.4	3.19 (1.29)	3.09–3.28	16	0.4	3.04 (1.78)	2.16–3.91
Cardiovascular and diabetes	723	1.4	3.04 (1.30)	2.95–3.13	23	0.5	3.48 (2.08)	2.63–4.33
Other prescriptions	19,715	37.9	3.63 (1.36)	3.61–3.65	1496	37.4	3.48 (1.54)	3.40–3.56
No prescription data	25,644	49.3	3.78 (1.48)	3.76–3.79	2184	54.6	3.50 (1.74)	3.43–3.58
30–39 y								
All users	79,508	33.0	3.74 (1.53)	3.73–3.75	6826	100	3.78 (1.78)	3.74–3.82
Female sex	52,807	66.4	3.61 (1.46)	3.60–3.62	3820	56.0	3.67 (1.73)	3.61–3.72
Male sex	12,486	15.7	4.04 (1.67)	4.02–4.07	1274	18.7	4.06 (2.00)	3.95–4.17
Unstated sex	14,215	17.9	3.95 (1.63)	3.92–3.98	1732	25.4	3.82 (1.70)	3.74–3.90
Cardiovascular	11,677	14.7	3.41 (1.42)	3.38–3.43	676	9.9	3.57 (1.62)	3.45–3.69
Diabetes	1252	1.6	3.29 (1.35)	3.21–3.36	33	0.5	3.16 (1.42)	2.67–3.64
Cardiovascular and diabetes	2506	3.2	3.04 (1.31)	2.99–3.09	63	0.9	3.61 (1.66)	3.20–4.01
Other prescriptions	27,296	34.3	3.73 (1.48)	3.71–3.75	2309	33.8	3.74 (1.72)	3.67–3.81
No prescription data	36,777	46.2	3.92 (1.60)	3.90–3.93	3745	54.9	3.85 (1.85)	3.79–3.91
40–49 y								
All users	58,406	24.2	3.75 (1.65)	3.73–3.76	4373	100	3.84 (1.72)	3.79–3.89
Female sex	38,373	65.7	3.63 (1.58)	3.61–3.65	2300	52.6	3.64 (1.63)	3.57–3.71
Male sex	10,508	18.0	4.02 (1.77)	3.99–4.05	1055	24.1	4.20 (1.90)	4.09–4.32
Unstated sex	9525	16.3	3.91 (1.73)	3.88–3.95	1018	23.3	3.93 (1.63)	3.83–4.03
Cardiovascular	11,981	20.5	3.45 (1.53)	3.43–3.48	673	15.4	3.75 (1.84)	3.61–3.89
Diabetes	608	1.0	3.31 (1.49)	3.19–3.43	23	0.5	3.31 (1.13)	2.84–3.77
Cardiovascular and diabetes	3017	5.2	3.07 (1.47)	3.01–3.12	72	1.6	3.37 (1.29)	3.07–3.67
Other prescriptions	16,240	27.8	3.78 (1.58)	3.75–3.80	1277	29.2	3.85 (1.67)	3.76–3.94
No prescription data	26,560	45.4	3.95 (1.72)	3.93–3.97	2328	53.2	3.89 (1.72)	3.81–3.96
50–59 y								
All users	35,093	14.6	3.77 (1.76)	3.75–3.79	1641	100	3.69 (1.73)	3.61–3.77
Female sex	23,424	66.7	3.66 (1.69)	3.64–3.68	870	53.0	3.52 (1.70)	3.41–3.63
Male sex	6251	17.8	4.06 (1.88)	4.02–4.11	440	26.8	4.04 (1.86)	3.86–4.21
Unstated sex	5418	15.4	3.92 (1.88)	3.87–3.97	331	20.2	3.67 (1.56)	3.50–3.84
Cardiovascular	8756	25.0	3.53 (1.62)	3.50–3.56	342	20.8	3.63 (1.67)	3.45–3.80
Diabetes	255	0.7	3.26 (1.45)	3.08–3.44	6	0.4	2.17 (1.19)	1.22–3.12
Cardiovascular and diabetes	2285	6.5	3.02 (1.51)	2.96–3.08	60	3.7	3.15 (1.43)	2.79–3.52
Other prescriptions	7800	22.2	3.85 (1.74)	3.81–3.89	387	23.6	3.90 (1.86)	3.72–4.09
No prescription data	15,997	45.6	3.98 (1.84)	3.95–4.01	846	51.6	3.67 (1.71)	3.55–3.78
60+ y								
All users	15,938	6.6	3.55 (1.82)	3.52–3.58	475	100	3.56 (1.79)	3.40–3.72
Female sex	9707	60.9	3.38 (1.73)	3.35–3.42	214	45.1	3.48 (1.77)	3.24–3.72
Male sex	3831	24.0	3.88 (1.93)	3.81–3.94	189	39.8	3.76 (1.94)	3.48–4.04
Unstated sex	2400	15.1	3.70 (1.88)	3.62–3.77	72	15.2	3.28 (1.37)	2.96–3.60
Cardiovascular	4867	30.5	3.34 (1.73)	3.29–3.39	129	27.2	3.58 (1.92)	3.25–3.92
Diabetes	79	0.5	3.18 (1.48)	2.85–3.50	–	–	–	–
Cardiovascular and diabetes	1271	8.0	2.82 (1.55)	2.73–2.90	25	5.3	2.97 (1.60)	2.35–3.60
Other prescriptions	2961	18.6	3.68 (1.76)	3.62–3.75	101	21.3	3.81 (1.79)	3.46–4.16
No prescription data	6760	42.4	3.78 (1.90)	3.74–3.83	220	46.3	3.50 (1.73)	3.27–3.73

CI: confidence interval; SD standard deviation.

Overall, our study participants were less likely to have been treated for diabetes (5.3% versus 9.1%) and cardiovascular (21.8% versus 37.8%)^24^ than the general population aged 20 and over. We conducted subgroup analyses to determine whether condition rates varied by age. Our younger adult participants aged 20–39 were treated for cardiovascular conditions (14.7%) and diabetes (3.65%) at higher rates compared to the prevalence of these conditions among Americans aged 20–39 (12.5% and 2.6%, respectively).^25,26^ On the other hand, middle-aged (40–59) and older (60+) adults in our study were treated for cardiovascular conditions (middle-aged = 28.6% versus 40.4%; older = 38.5% versus 77.35%) and diabetes (middle-aged = 6.7% versus 12.7%; older = 8.5% versus 20.8%) at lower rates compared to the population-based prevalence of these conditions among Americans in the same age groups.^25,26^

### Daily walk and run distances

Study participants walked and ran a mean of 3.72 miles per day. A one-way ANOVA F-test showed a statistically significant difference in the activity levels of different age groups, F(4, 241009) = 80.14, *p *<* *0.001. On average, younger participants recorded higher daily walk distances than older participants. Post hoc comparisons using Tukey’s honestly significant difference (HSD) tests indicated that walk distances did not significantly differ between those in the 40–49 and 50–59 age brackets, while all other pairs’ differences were found to be statistically significant. 

A one-way ANOVA F-test revealed a statistically significant difference in mean daily walk and run distances by condition status, F(1, 129273) = 1934.60, *p *<* *0.001, demonstrating significant differences in activity levels among participants whose pharmacy data showed treatment for at least one of the two identified chronic conditions compared to those with neither. Post hoc comparisons using Tukey’s HSD tests found all pairs of groups to be significantly different.

**Figure 1.**
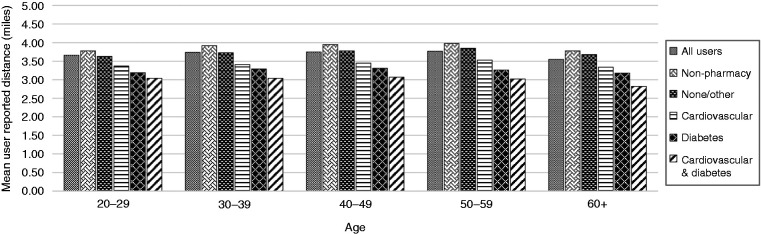
Mean distances walked and/or run each day, stratified by age and by chronic conditions (cardiovascular disease (CVD) and/or diabetes) being managed by the participant.

A one-way ANOVA F-test showed a statistically significant difference in mean daily walk and run distances between groups of pharmacy customers treated for cardiovascular conditions and diabetes, other pharmacy customers, and non-pharmacy customers, F(4, 241009) = 1231.93, *p *<* *0.001. Post hoc comparisons using Tukey’s HSD tests found all pairs of groups to be significantly different (Figure 1).

Risk ratios determined whether participants treated with cardiovascular medications, diabetes medications or both were less likely to be sufficiently active (mean distance per day ≥3.75 miles) than those who were not treated for either condition. Participants fitting our definition of being sufficiently active (*n *=* *50,965) made up 39.42% of the total participants with any pharmacy data. Analysis of covariance showed that condition treatment was associated with physical activity (cardiovascular only: F(1, 116564) = 707.86, *p *<* *0.0001; diabetes only: F(1, 76909) = 201.86, *p *<* *0.0001; both cardiovascular and diabetes: F(1, 83808) = 1282.80, *p *<* *0.0001) when including age as a covariate in the model (cardiovascular only: F(1, 116564) = 47.42, *p *<* *0.0001; diabetes only: F(1, 76909) = 48.46, *p *<* *0.0001; both cardiovascular and diabetes: F(1, 83808) = 40.40, *p *<* *0.0001). An interaction between age and condition treatment was also significant for cardiovascular only and both cardiovascular and diabetes (F(1, 116564) = 7.44, *p *=* *0.0064 and F(1, 83808) = 9.26, *p *=* *0.0023 respectively), but not for diabetes only (F(1, 76909) = 0.26, *p *=* *0.6086).

**Figure 2.**
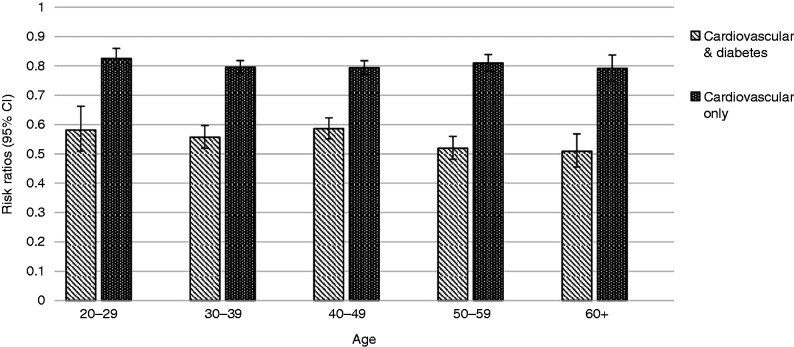
Risk ratios for condition category and obtaining sufficient physical activity by age group. Each condition category was compared to pharmacy patients who did not have an indication for cardiovascular disease (CVD) or diabetes. Sufficient physical activity is a mean distance ≥3.75 miles per day.

The binomial regression models demonstrated associations between each condition category (cardiovascular only, diabetes only and both cardiovascular and diabetes) and obtaining sufficient physical activity. Participants treated for diabetes only were significantly less likely (70.41%, 95% CI 66.61–74.42%) to be sufficiently active than users who were not being treated for cardiovascular or diabetes. Cardiovascular only and both cardiovascular and diabetes regression models were stratified on 10-year age groups due to the interaction between condition treatment and age. Those treated with cardiovascular medications only were less likely to be sufficiently active than participants who were not being treated for cardiovascular conditions or diabetes at each age group (age 20–29: 82.50%, 95% CI 79.17–85.96%; age 30–39: 79.57%, 95% CI 77.38–81.82%; age 40–49: 79.43%, 95% CI 77.16–81.78%; age 50–59: 80.99%, 95% CI 78.19–83.89%; age 60+: 79.14%, 95% CI 74.81–83.72%). Compared with diabetes only and cardiovascular only, there was a stronger effect of treatment with both cardiovascular and diabetes medications on being less likely to be sufficiently active (age 20–29: 58.10%, 95% CI 50.94–66.26%; age 30–39: 55.68%, 95% CI 51.92–59.72%; age 40–49: 58.59%, 95% CI 55.11–62.29%; age 50–59: 51.92%, 95% CI 48.16–55.97%; age 60+: 50.86%, 95% CI 45.54–56.80%). Figure 2 demonstrates the interaction effect after stratifying by age category and treatment with cardiovascular medications only and both cardiovascular and diabetes medications.

## Discussion

Study participants walked on average 3.72 miles per day, which is equivalent to 7440 steps, approximately on target for meeting the recommended daily number of 7000–8000 steps.^23^ Consistent with other studies, our younger participants recorded higher daily walk distances than older participants.^27^

Overall, our study participants were less likely to be treated with cardiovascular and diabetes medications compared to the rates of US adults aged 20 and over with these conditions.^25,26^ However, the rates across age groups tell a different story. Our younger adult participants aged 20–39 were treated with cardiovascular and diabetes medications at higher rates compared to the prevalence of these conditions among Americans of the same age. On the other hand, middle-aged (40–59) and older (60+) adults in our study were treated with cardiovascular and diabetes medications at lower rates compared to the prevalence of these conditions among Americans in the same age group's.^24–26^ These differences between our cohort and the US population-based rates are indicative of the differences attributable to the self-selection of individuals into this particular program. More specifically, program participants sought out BRhc, own and regularly use a fitness tracker, and are sufficiently technologically literate to enable connection of the fitness tracker within the BRhc. Based on these attributes, one might expect to find socioeconomic differences between the US population and the program participants; however, the differences in rates of cardiovascular and diabetes conditions were not expected.

Marshall et al. estimated that 3000 steps approximately equates to 30 minutes of activity, although one would expect the total daily step count to greatly exceed 3000 steps for an active individual as cumulative movement throughout the day is captured.^28^ Tudor-Locke et al. estimated that a total daily step count of 7000–8000 steps, or 3.5–4.0 miles per day, would satisfy current CDC moderate/vigorous activity recommendations.^23^ On average, participants across all age groups in our study met daily physical activity recommendations according to the Tudor-Locke approach. The recommended daily step count for older adults, 7100 steps per day, is minimally different from that recommended for adults of any age.^23^ The mean walk distance of our older participants was 3.55 miles (7100 steps) per day. Activity levels of our older participants are exceptionally high; on average 22.6% of older adults engage in the recommended amount of daily moderate/vigorous physical activity.^29^

Adults who track daily steps are more likely to increase their physical activity.^30^ Population-level physical activity rates show that approximately 36% of adults with diabetes and 40.1% of adults with cardiovascular meet physical activity recommendations.^14^ Participants who were treated for cardiovascular and/or diabetes conditions were significantly less likely to be sufficiently active than participants who were not being treated for either condition. Nonetheless, the mean distance walked per day for adults treated with cardiovascular medications was 3.43 miles and 3.26 miles for adults treated with diabetes medications, indicating that participants being treated for these conditions are more active than participants in the general US population with these conditions. As expected, activity levels decreased for participants who were being treated for *both* cardiovascular condition and diabetes. Participants treated for both conditions walked nearly 1 mile less each day than patients who did not have a prescription fill during the study period (2.97 versus 3.93 miles).

### Limitations

This study analyzed data derived from a large database of consumer-grade activity trackers. While the amount and source of the data contribute to the novelty of this research, these attributes are also limitations. Randomization was not possible; our participants self-selected to enroll in the BRhc program, making our cohort less generalizable to the US population. Several noteworthy differences were observed between our study participants and general population rates, which could be due to self-selection bias. Our study participants may be more likely to be physically active and self-manage their health conditions relative to the general population with similar age and disease-state attributes. Moreover, individuals who track behavior (i.e. physical activity) are more likely to achieve the desired behavior than those who do not track,^21^ making participants in this study even more likely to meet physical activity recommendations.

Only objective data recorded with wearable devices were included in the analysis, since subjective measures from self-reported data were not trustable, potentially introducing an additional bias. Approximately 16% of objective records were excluded in order to produce an analytic data set of the highest quality; however, the data exclusions can also be construed as a limitation. We are aware of the limitations associated with using the BRhc dataset, but we also see this as an opportunity to explore a new way of doing research in the age of “big data.” While there are no doubts that consumer-grade fitness trackers are imperfect for research assessment at this time, the field will move forward by expanding the research base that relies on these technologies, providing a better understanding of the conditions for cleaning and interpreting these data. Despite these limitations, the BRhc participant data presents a unique opportunity to validate and expand upon previous literature by examining physical activity rates across a large population of American adults with chronic conditions.

In addition to addressing these limitations, recommendations for future research include examining the impact of varying levels of financial incentives on physical activity participation. Moreover, another potential next step includes referral of insufficiently active BRhc participants treated for chronic conditions into a structured, condition-specific evidence-based physical activity behavior change program to determine whether such programs significantly increase activity participation beyond the increase attributable to BRhc.

## Conclusion

BRhc is a nationally disseminated, incentive-driven effort to increase physical activity participation among adults in the United States, including those being treated for cardiovascular conditions and diabetes. Benefits of digital health programs, but specifically BRhc, include their broad reach amongst US adults, the strong potential for the program’s sustainability given the stability and consistency of the organization’s infrastructure, and the use of digital technology for automatic activity tracking coupled with incentives to encourage participant behavioral maintenance.^31^

This study reinforced previous findings regarding physical activity and common chronic conditions. We found that participants treated for cardiovascular conditions and/or diabetes were less likely to be sufficiently active than participants who were not being treated for either condition. Through its digital format, BRhc efficiently reaches a large number of Americans in an effort to keep them engaged over long periods of time.

## Authors' note

Jenny Z Jiang is now affiliated with Center for Observational Research and Data Sciences, Bristol-Myers Squibb, Lawrenceville, New Jersey, USA.
